# Supplementary material for: Complex‐centric proteome profiling by SEC‐SWATH‐MS
Source: Mol Syst Biol. 2019 Jan 14;15(1):e8438. doi: 10.15252/msb.20188438 (PMC6346213; doi:10.15252/msb.20188438)
Supplement: Supplementary file 7 — Dataset EV6 [file MSB-15-e8438-s007.zip › feature_plots_bioplex/Q9BYG3.pdf]

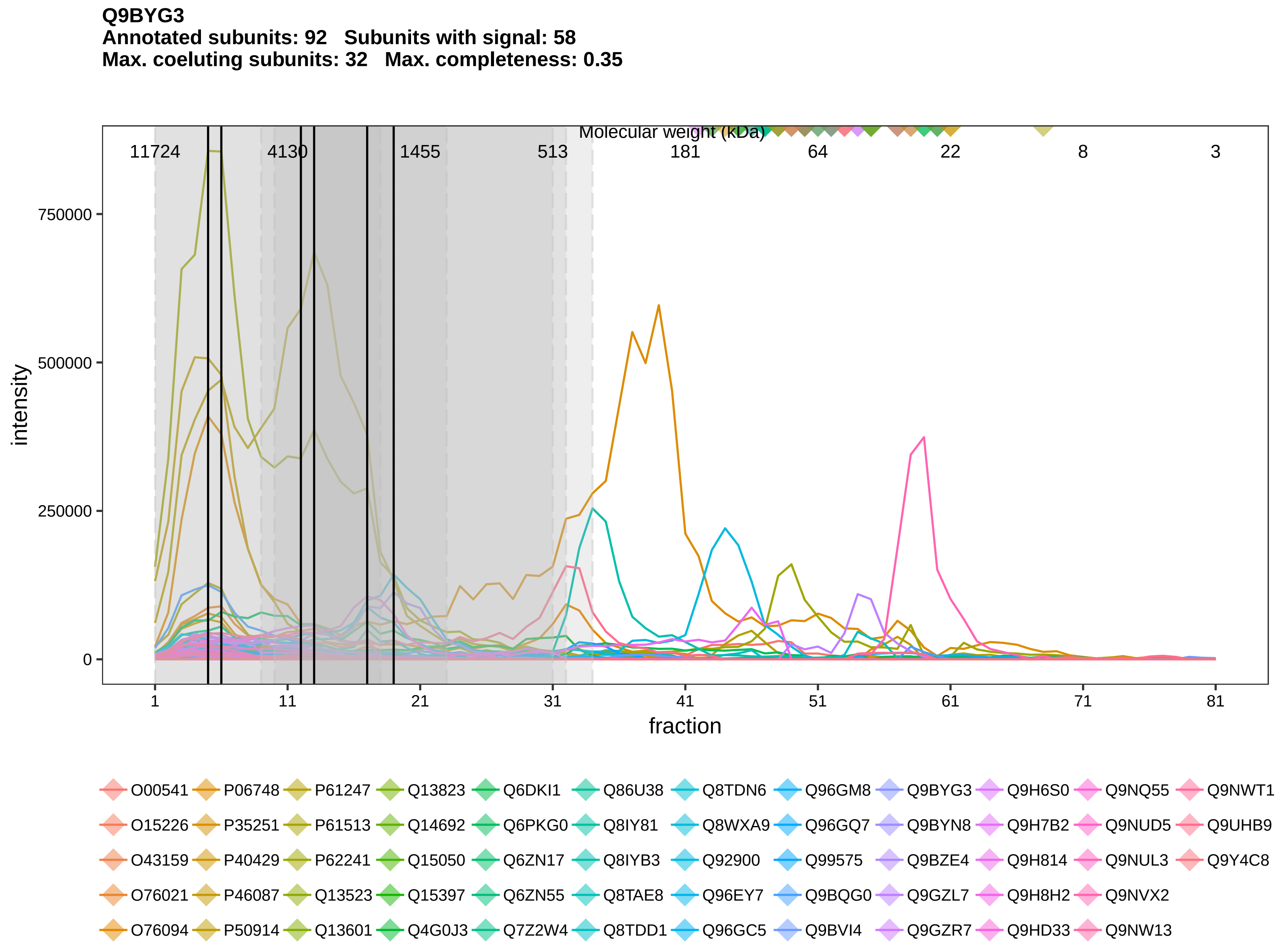
Q9BYG3
Annotated subunits: 92 Subunits with signal: 58
Max. coeluting subunits: 32 Max. completeness: 0.35
Molecular weight (kDa)
11724
4130
1455
513
181
64
22
8
3
intensity
750000
500000
250000
0
fraction
1
11
21
31
41
51
61
71
81
O00541
P06748
P61247
Q13823
Q6DKI1
Q86U38
Q8TDN6
Q96GM8
Q9BYG3
Q9H6S0
Q9NQ55
Q9NWT1
O15226
P35251
P61513
Q14692
Q6PKG0
Q8IY81
Q8WXA9
Q96GQ7
Q9BYN8
Q9H7B2
Q9NUD5
Q9UHB9
O43159
P40429
P62241
Q15050
Q6ZN17
Q8IYB3
Q92900
Q99575
Q9BZE4
Q9H814
Q9NUL3
Q9Y4C8
O76021
P46087
Q13523
Q15397
Q6ZN55
Q8TAE8
Q96EY7
Q9BQG0
Q9GZL7
Q9H8H2
Q9NVX2
O76094
P50914
Q13601
Q4G0J3
Q7Z2W4
Q8TDD1
Q96GC5
Q9BVI4
Q9GZR7
Q9HD33
Q9NW13
